# Performance changes due to differences among annotating radiologists for training data in computerized lesion detection

**DOI:** 10.1007/s11548-024-03136-9

**Published:** 2024-04-16

**Authors:** Yukihiro Nomura, Shouhei Hanaoka, Naoto Hayashi, Takeharu Yoshikawa, Saori Koshino, Chiaki Sato, Momoko Tatsuta, Yuya Tanaka, Shintaro Kano, Moto Nakaya, Shohei Inui, Masashi Kusakabe, Takahiro Nakao, Soichiro Miki, Takeyuki Watadani, Ryusuke Nakaoka, Akinobu Shimizu, Osamu Abe

**Affiliations:** 1 Center for Frontier Medical Engineering, Chiba University, 1-33 Yayoi-cho, Inage-ku, Chiba, 263-8522 Japan; 2 Department of Computational Diagnostic Radiology and Preventive Medicine, The University of Tokyo Hospital, Tokyo, Japan; 3 Department of Radiology, The University of Tokyo Hospital, Tokyo, Japan; 4 Division of Radiology and Biomedical Engineering, Graduate School of Medicine, The University of Tokyo, Tokyo, Japan; 5 Department of Radiology, Tokyo Metropolitan Bokutoh Hospital, Tokyo, Japan; 6 Department of Diagnostic Radiology, Kitasato University Hospital, Sagamihara, Kanagawa Japan; 7 Department of Radiology, NTT Medical Center Tokyo, Tokyo, Japan; 8 Division of Medical Devices, National Institute of Health Sciences, Kawasaki, Kanagawa Japan; 9 Institute of Engineering, Tokyo University of Agriculture and Technology, Tokyo, Japan

**Keywords:** Computer-aided detection (CAD), Machine learning, Retraining, Annotation

## Abstract

**Purpose:**

The quality and bias of annotations by annotators (e.g., radiologists) affect the performance changes in computer-aided detection (CAD) software using machine learning. We hypothesized that the difference in the years of experience in image interpretation among radiologists contributes to annotation variability. In this study, we focused on how the performance of CAD software changes with retraining by incorporating cases annotated by radiologists with varying experience.

**Methods:**

We used two types of CAD software for lung nodule detection in chest computed tomography images and cerebral aneurysm detection in magnetic resonance angiography images. Twelve radiologists with different years of experience independently annotated the lesions, and the performance changes were investigated by repeating the retraining of the CAD software twice, with the addition of cases annotated by each radiologist. Additionally, we investigated the effects of retraining using integrated annotations from multiple radiologists.

**Results:**

The performance of the CAD software after retraining differed among annotating radiologists. In some cases, the performance was degraded compared to that of the initial software. Retraining using integrated annotations showed different performance trends depending on the target CAD software, notably in cerebral aneurysm detection, where the performance decreased compared to using annotations from a single radiologist.

**Conclusions:**

Although the performance of the CAD software after retraining varied among the annotating radiologists, no direct correlation with their experience was found. The performance trends differed according to the type of CAD software used when integrated annotations from multiple radiologists were used.

## Introduction

**Fig. 1:**
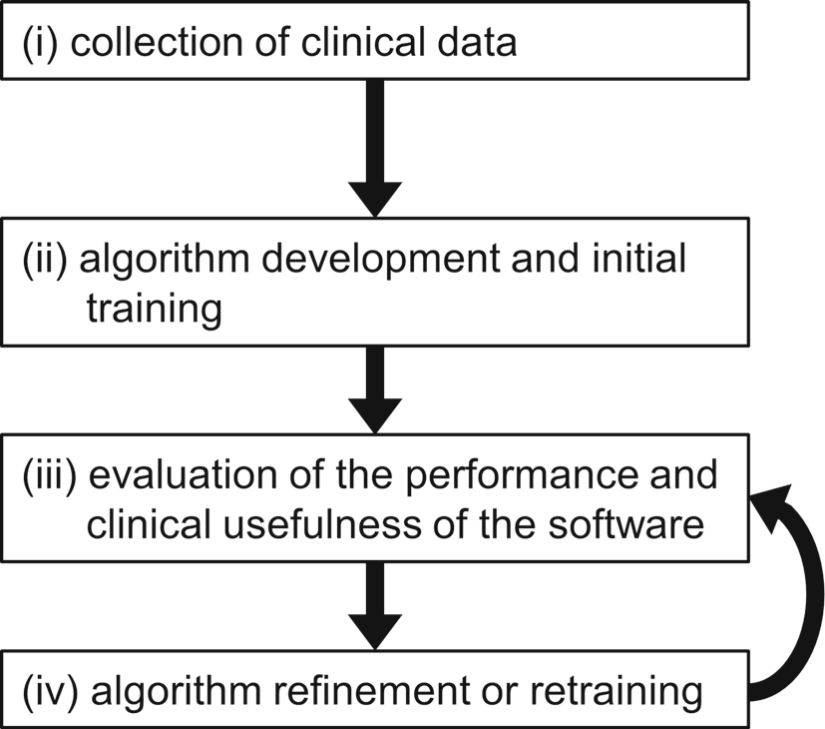
Process for development of CAD software

Computer-aided detection (CAD) software has been developed by numerous research groups, and CAD software using machine learning, particularly deep learning, has increased in recent years [1–5]. The development of CAD software based on machine learning involves: (i) the collection of clinical data, (ii) algorithm development and initial training, (iii) the evaluation of the performance and clinical usefulness of the software, and (iv) algorithm refinement or retraining (Fig. 1) [6]. This development is not a one-time process, even for commercially available CAD software, and it involves repeated cycles of steps (iii) and (iv). The performance of the CAD software depends on the quality and quantity of the datasets used for machine learning. If the data characteristics differ between development and practical use, the performance of the CAD software degrades. The main factors causing changes in the performance of CAD software are as follows.

1. Difference in subject populations
2. Difference in scanners or scan parameters
3. Quality and bias of annotation by annotators (e.g., radiologists)

**Fig. 2:**
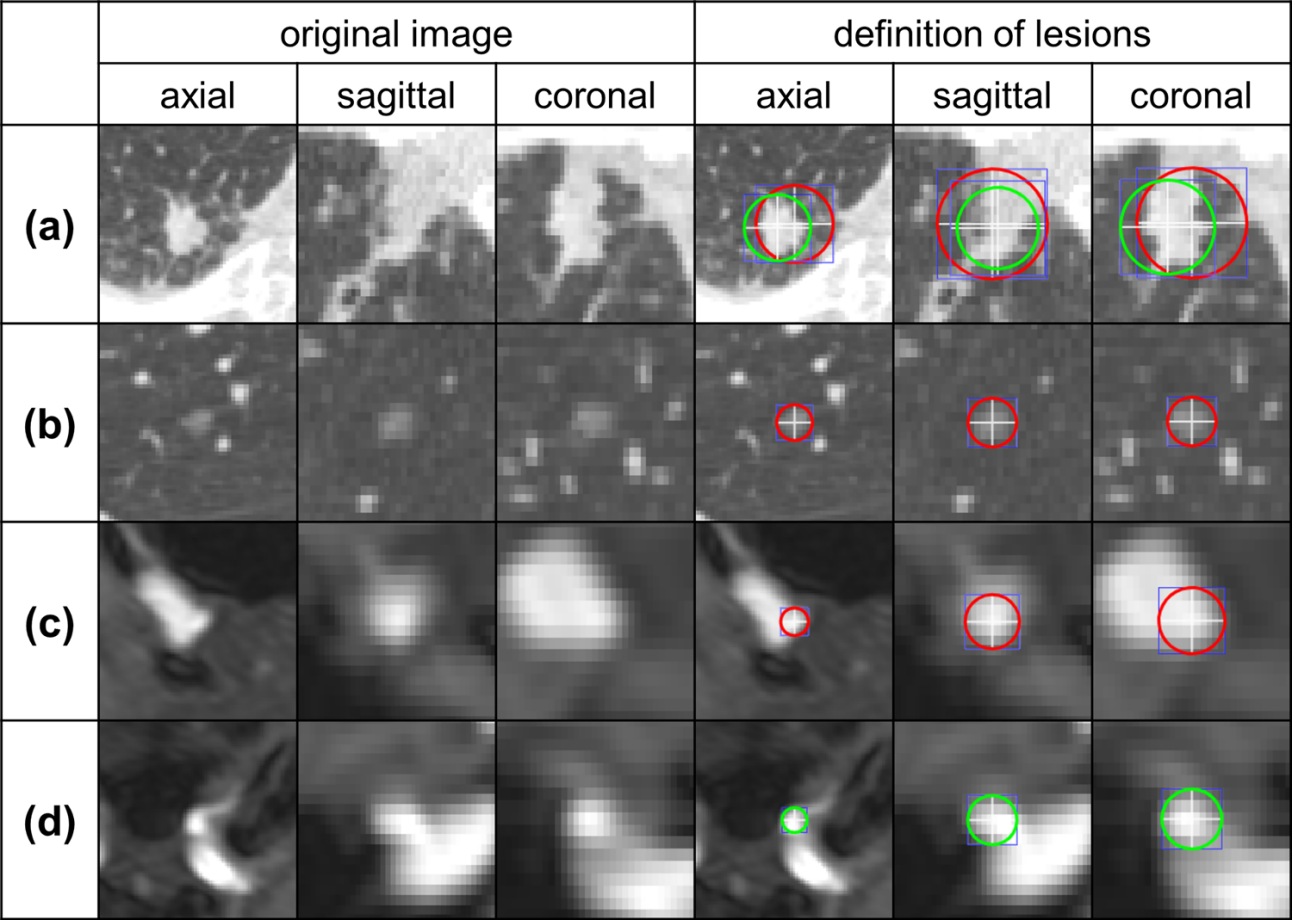
Examples of mismatch in annotations of spherical ROI by two radiologists. **a** 15.8 mm solid nodule, annotated by both, but with differing ranges. **b** 6.6 mm pure ground-glass nodule, annotated by only one radiologist. **c** 3.2 mm saccular aneurysm, annotated by only one radiologist. **d** Infundibular dilation at the origin of the left ophthalmic artery, incorrectly annotated as a saccular aneurysm by one radiologist

Several research groups, including ours, have reported changes in CAD software performance caused by the first two factors [7–14]. These changes can be overcome through continuous data collection and retraining [9, 10, 14]. Regarding Factor (3), for example, in the case of the Lung Image Database Consortium–Image Database Resource Initiative (LIDC-IDRI) database of lung nodules in chest computed tomography (CT) images [15], the diagnoses and defined contours vary among annotating radiologists [16]. Tachibana et al. reported that incorporating information about radiologists who performed annotations in the classification of brain aneurysms in magnetic resonance angiography (MRA) images improved classification performance [17]. Figure 2 shows the examples of annotation variability in spherical regions of interest (ROIs) by two radiologists. These annotations encompassed the entire lesion in three dimensions (3D). We hypothesized that a potential factor contributing to this variability is the difference in years of experience among radiologists in image interpretation. Moreover, integrating annotations from multiple radiologists may reduce variability among annotators. However, to the best of our knowledge, no studies have focused on either the years of experience in image interpretation among annotators or the relationship between methods of annotation integration and the performance of CAD software.

In this study, we investigated the following two items regarding the change in performance in the retraining of CAD software owing to the years of experience in image interpretation among radiologists who perform annotation work.

- Whether a relationship was observed between the years of experience of the annotating radiologists and the performance of the trained CAD software.
- Whether the performance of the CAD software is improved by integrating annotations from multiple radiologists with different years of experience.

We targeted two types of CAD software for lung nodule detection in chest CT images and cerebral aneurysm detection in MRA images, without altering Factors (1) and (2) mentioned above.

## Materials and methods

### Datasets

**Fig. 3:**
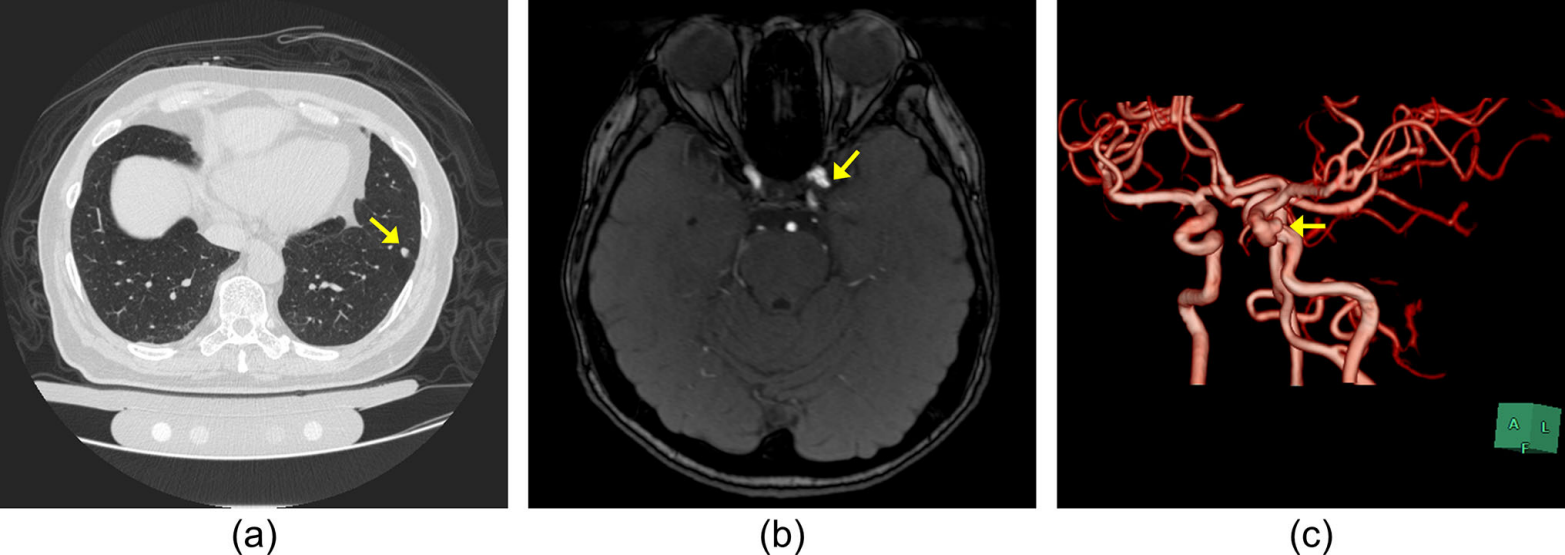
Examples of target lesions included in the test set. **a** Lung nodule (8.9 mm solid nodule) in chest CT images, **b** cerebral aneurysm (4.3 mm in left internal carotid artery) in brain MRA images, **c** pre-rendered whole-brain volume rendering images of the same case as in (**b**). A yellow arrow indicates the target lesion, but the arrows are not visible during actual reading

**Fig. 4:**
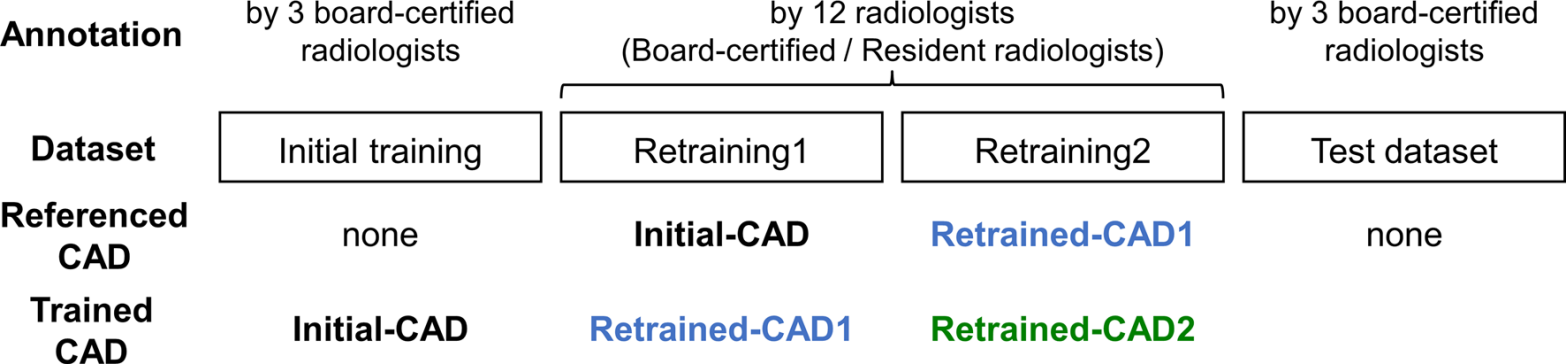
Procedure of annotation of dataset and training for each CAD software

This retrospective study was approved by the Ethics Review Board of our institution. We utilized a chest CT dataset for lung nodule detection (Fig. 3a) and a brain MRA dataset for cerebral aneurysm detection (Fig. 3b) collected from our institution. The subjects were adults who underwent annual whole-body general medical examinations, including chest CT or brain MRA. Written informed consent was obtained from all participants to use their clinical images for research. Each dataset consisted of subsets for the initial training, two sets for retraining (Retraining1 and Retraining2), and a test, as shown in Fig. 4. Table 1 shows the number of cases in the chest CT and brain MRA datasets that are common to both datasets. The criteria for selecting the positive and negative cases are described in the following subsections. In the retraining and test sets, 10 ambiguous cases were used as negative cases.

**Table 1:** Number of cases for chest CT and brain MRA datasets (common to both datasets)

	Initial training	Retraining1	Retraining2	Test
Positive cases	50	40	40	50
Negative cases	0	35*	35*	50*
Total	50	75	75	100

*Ten cases included lung nodules < 6 mm in diameter in the chest CT dataset and infundibular dilation or basilar artery bifurcation in the brain MRA dataset

### Chest CT dataset

**Fig. 5:**
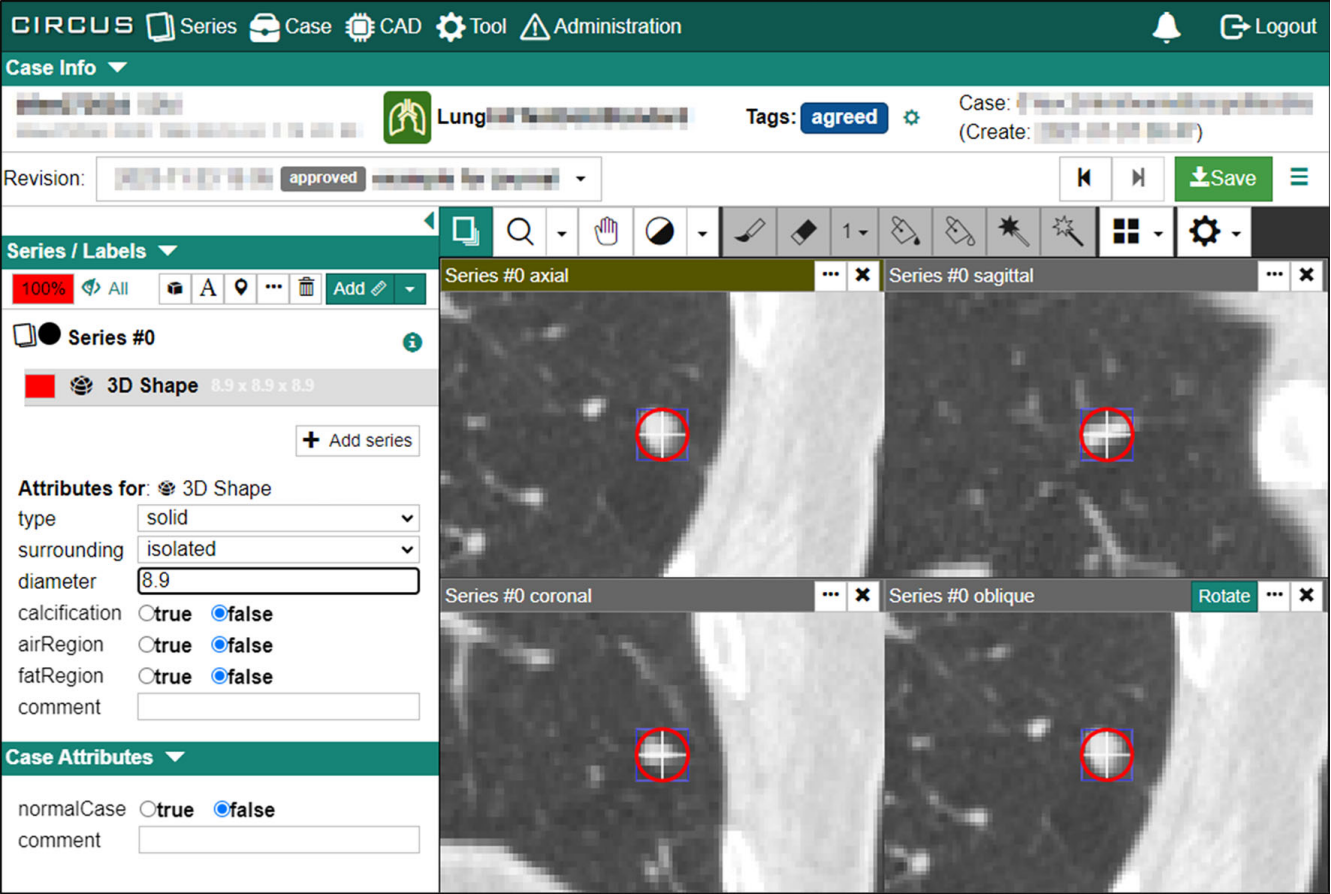
Web interface for defining spherical ROI in CIRCUS DB. The left panel has a series selector and an attribute editor. The right panel has a grid of DICOM viewer components, which includes an axial view, a sagittal view, a coronal view, and an oblique view

We used a total of 300 cases of chest CT images. The datasets were acquired from a GE LightSpeed CT scanner (GE Healthcare, Waukesha, WI, USA). The original voxel size was 0.781 × 0.781 × 1.250 mm^3^. The acquisition parameters were as follows: number of detector rows, 16; tube voltage, 120 kVp; tube current, 50–290 mA (automatic exposure control); noise index, 20.41; rotation time, 0.5 s; moving table speed, 70 mm/s; body filter, standard; reconstruction slice thickness and interval, 1.25 mm; field of view, 400 mm; matrix size, 512 × 512 pixels; pixel spacing, 0.781 mm. Each positive case included at least one lung nodule with a diameter of 6 mm or more. Two board-certified radiologists (N.H. and S.H., with 32 and 19 years of experience in chest CT interpretation, respectively) annotated the cases in the initial training and test subsets via spherical ROIs to encompass the entire nodule in 3D using the web-based image database system CIRCUS DB [6] (Fig. 5). Discrepancies between the two radiologists were resolved by a third board-certified radiologist (T.W., with 20 years of experience). The cases for retraining subsets were selected based on radiology reports by consensual reading by two experienced radiologists. Ten negative cases in the retraining and test subsets included pulmonary nodules of < 6 mm in diameter.

### Brain MRA dataset

We used a total of 300 cases of brain MRA images. These images were acquired using four 3-Tesla MR scanners (two Signa HDxt, GE Healthcare, Waukesha, WI, USA; one Discovery MR750, GE Healthcare; and one Skyra, Siemens Healthcare, Erlangen, Germany). Table 2 presents the details of the examination. Each positive case included at least one saccular aneurysm with a diameter of 2 mm or more. Two board-certified radiologists (N.H. and S.M., with 32 and 14 years of experience in MRA interpretation, respectively) annotated the cases in the initial training and test subsets via spherical ROIs to encompass the entire aneurysm in 3D using the CIRCUS DB. Discrepancies between the two radiologists were resolved by a third board-certified radiologist (T.Y., with 26 years of experience). The cases for the retraining subsets were selected based on radiology reports by consensual reading by two experienced radiologists. Ten of the negative cases in the retraining and test subsets included infundibular dilation (Fig. 1d) or basilar artery bifurcation.

**Table 2:** Specification of the brain MRA datasets

MR scanners	Scan parameters
Two Signa HDxt and one Discovery MR750 (GE Healthcare, Waukesha, WI, USA)	Field of view (FOV), 240 mm; matrix size, 512 × 512 pixels; pixel spacing, 0.469 mm; slice thickness, 1.2 mm; slice interval, 0.6 mm; repetition time (TR), 22 or 25 ms; echo time (TE), 2.7–3.3 ms; flip angle, 15°
Skyra (Siemens Healthcare, Erlangen, Germany)	FOV, 230 mm; percent phase field of view, 82.3%; matrix size, 768 × 632 pixels; pixel spacing, 0.299 mm; slice thickness, 0.6 mm; slice interval, 0.6 mm; TR, 20 ms; TE, 3.69 ms; flip angle, 13°

## CAD algorithm

### Lung nodule detection in CT images

Chest CT images were resampled to a 1.0 mm isotropic voxel size using tricubic interpolation, and the lung volume was extracted using the method described in [18]. Nodule candidates were extracted using volumetric curvature-based thresholding and region growing [19]. Subsequently, for each nodule candidate, a 32 × 32 × 32 cubic volume of interest (VOI) was extracted around the center of gravity of the nodule candidate. The VOIs were fed into a classifier based on 3D ResNet-18 [20] to classify true nodules and false positives (FPs). The hyperparameters of the model were as follows: loss function, cross-entropy loss; optimizer, momentum stochastic gradient descent; learning rate, 1.0 × 10^−5^; momentum, 0.99; weight decay, 0.001; minibatch size, 8; number of epochs, 500. The numbers of negative (FP) and positive (true lung nodule) VOIs were equalized using data augmentation and undersampling to address the interclass imbalance in the training data. For each positive VOI, 29 augmented VOIs were generated by random shifts within ± 4 voxels on the *x*-, *y*-, and *z*-axes, random scaling in the range of [0.85, 1.15], and random rotation (0°/90°/180°/270°) in each of the axial, coronal, and sagittal planes. By contrast, negative VOIs were randomly undersampled such that the numbers of negative and positive VOIs were the same. Augmented positive VOIs and sampled negative VOIs were changed for each epoch.

### Cerebral aneurysm detection in MRA images

MRA images were resampled to a 0.469 mm isotropic voxel size using tricubic interpolation, and the signal intensity distributions of the images were standardized by global piecewise linear mapping [21]. The arterial region was extracted using the region growing-based method described in [22]. The voxel-based classifier based on the convolutional neural network (CNN) was employed at the voxels of the arterial region. The inputs of the CNN model were two-dimensional images, which were generated from a VOI around the target voxel by applying a maximum intensity projection algorithm. The CNN model consisted of two convolutional layers, two max-pooling layers, and two fully connected layers. The output layer was a single unit, and a logistic function was applied to the output to convert it into a positive probability (ranging from 0 to 1). The hyperparameters of the CNN model were set according to a random search, as previously reported [23]. The number of epochs was set to 10.

### Image annotation and retraining CAD

The procedure for annotation and retraining of each CAD software is shown (Fig. 4).

1. Initial-CAD is the CAD software trained using the initial training subset.
2. Twelve radiologists annotated the Retraining1 subset in each CAD software (Table 3). They were split into two groups: board-certified radiologists with more than 5 years of experience and resident radiologists with less experience. The annotation was performed using the CIRCUS DB (Fig. 5). Each lesion was defined as a spherical ROI circumscribing the entire lesion in 3D. Initially, annotation was performed without referencing the results from the CAD software. Subsequently, the annotations were revised by referring to the lesion candidates indicated by the Initial-CAD, which were displayed using spherical ROIs. In the annotation for cerebral aneurysm detection, pre-rendered whole-brain volume-rendered images (Fig. 3c) were also observed using the XTREK VIEW software (J-MAC system, Inc., Sapporo, Japan).
3. The CAD software retrained by adding the Retraining1 subset annotated by each radiologist is defined as “Retrained-CAD1,” resulting in 12 variations for each CAD software.
4. The Retraining2 subset was annotated similarly to Step 2), referencing Retrained-CAD1, which was trained using annotated data from each of the radiologists.
5. The CAD software retrained by adding the Retraining2 subset annotated by each radiologist is defined as “Retrained-CAD2,” resulting in 12 variations for each CAD software.

**Table 3:** Number of radiologists who annotated Retraining1 and Retraining2 subsets for each CAD software

CAD	Board-certified radiologists	Resident radiologists
Lung nodule detection	5^a^	7^b^
Cerebral aneurysm detection	4	8^c^

^a^Including T.Y., M.K

^b^Including S. Koshino, C.S., M.T., M.N

^c^Including S. Koshino, Y.T., S. Kano

The performance of each retrained CAD software was evaluated on the test subset. The model of each CAD software was implemented using Python 3.8.5 and PyTorch 1.8.0 [24]. Each model was trained on an NVIDIA DGX A100 server equipped with two AMD Rome 7742 processors (AMD Inc., Santa Clara, CA, USA), 2 TB of memory, and eight graphics processing units (GPUs) (A100 with 40 GB of memory, NVIDIA, Santa Clara, CA, USA). A single GPU was used to train the model.

### Integration of annotations from multiple radiologists

We also investigated the performance changes when retraining was performed using integrated annotations from multiple radiologists. For the Retraining1 subset, we integrated annotations as follows: using the product set of annotations from the two radiologists (AND), the sum set of annotations from the two radiologists (OR), and majority voting from the three radiologists (VOTING). VOTING uses annotations integrated as follows:

- integration by OR between the first and second radiologists
- integration by AND between the result of a) and the third radiologist

**Fig. 6:**
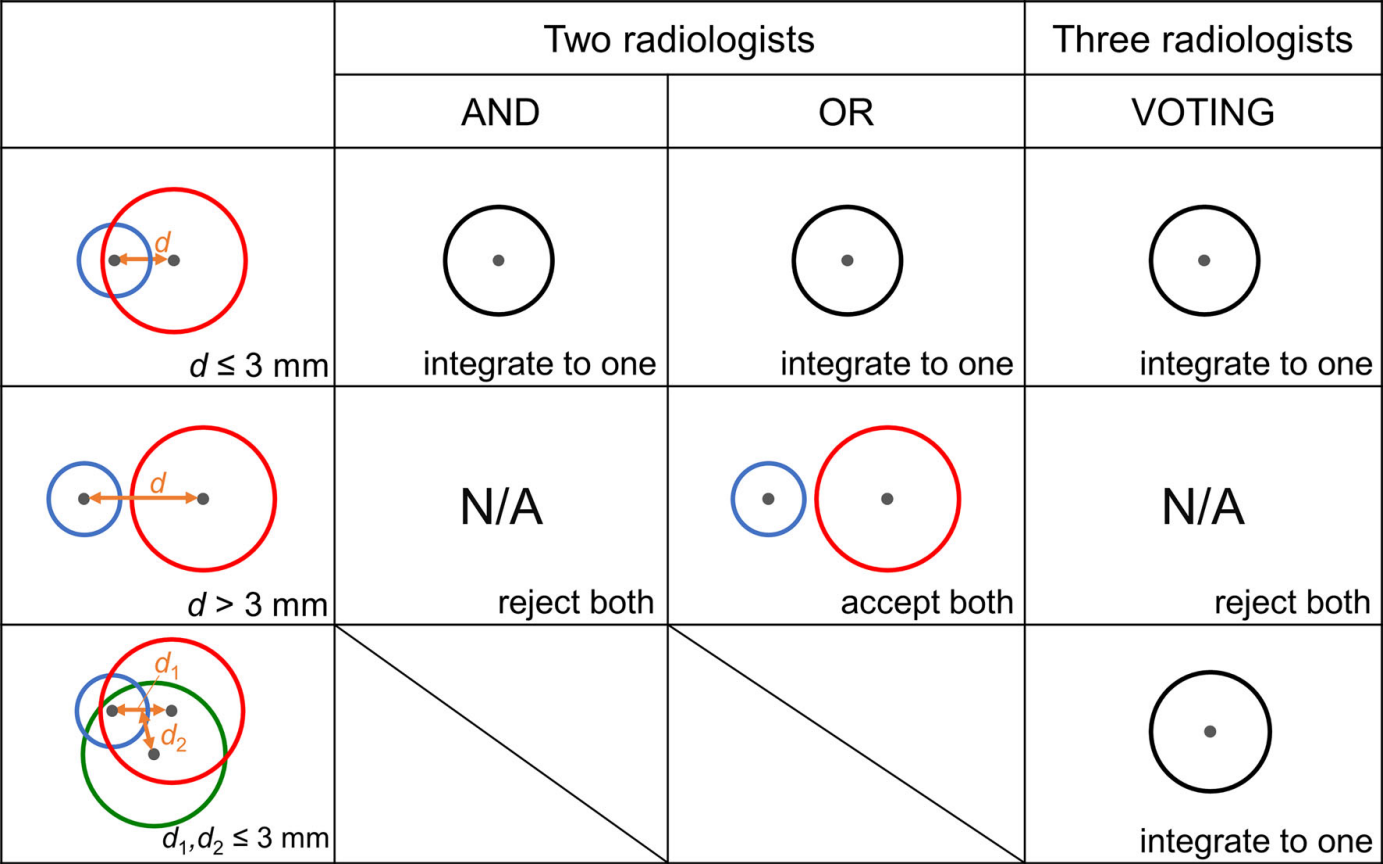
Examples of ways to integrate annotations among multiple radiologists (The color of circles differs among radiologists. Gray point: centroid of spherical ROI). In integrating annotations (black circle), the size and position of the center of gravity after integration were their averages

Figure 6 shows examples of ways to integrate annotations among multiple radiologists. We measured the distances between the centroids of spherical ROIs annotated by each radiologist exhaustively. If the distance between the centroids was within 3 mm, the annotations were integrated as the same, and the size and position of the centroid after integration were averaged. The measurements and integration were conducted automatically. The annotating radiologists selected were as follows: 12 radiologists (all radiologists), board-certified radiologists, and resident radiologists. Each CAD software retrained using integrated annotations was evaluated on the test subset.

The free-response operating characteristic (FROC) curve [25, 26], in which sensitivity is plotted against the number of FPs per case (FPs/case), is widely used to evaluate the performance of CAD software. To facilitate comparison between the FROC curves from different types of CAD software in a single number, the competition performance metric (CPM) [27], which defines the average sensitivity at predefined FPs/case (1/8, 1/4, 1/2, 1, 2, 4, and 8 FPs/case) along an FROC curve, was employed as the evaluation criterion. In the comparison between the integration strategy and CAD software, statistical analysis was conducted using the Steel–Dwass test, and a *p* value of less than 0.05 was considered statistically significant. Statistical analyses were performed using JMP Pro version 17.2.0 (JMP Statistical Discovery LLC, Cary, NC, USA).

## Results

**Fig. 7:**
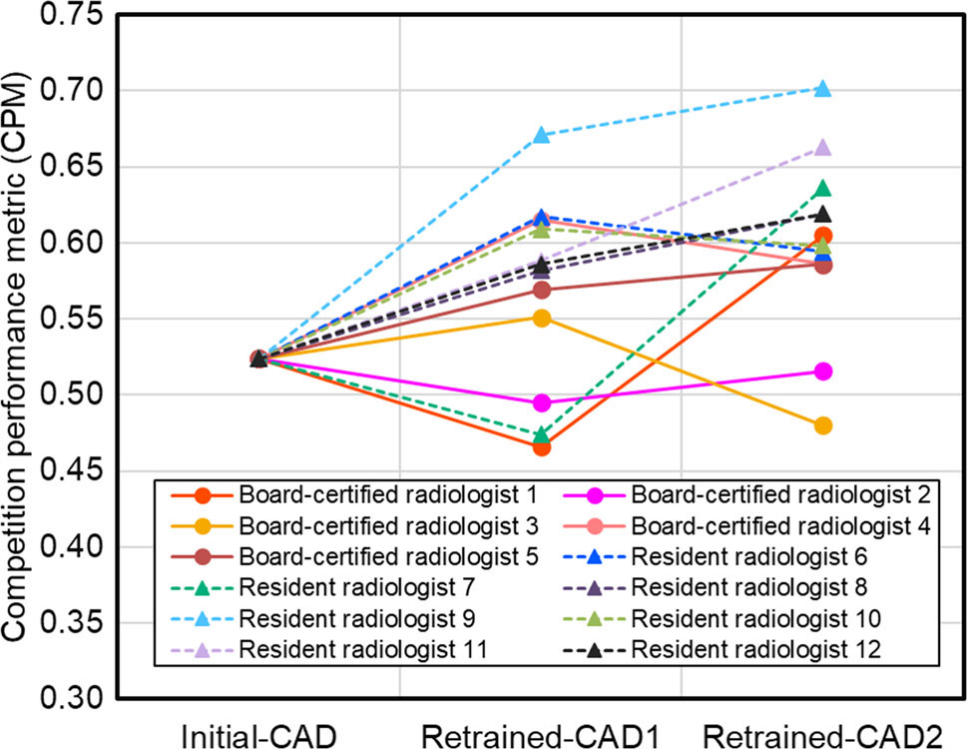
Performance change after retraining using the annotated data from each radiologist in the lung nodule detection

**Fig. 8:**
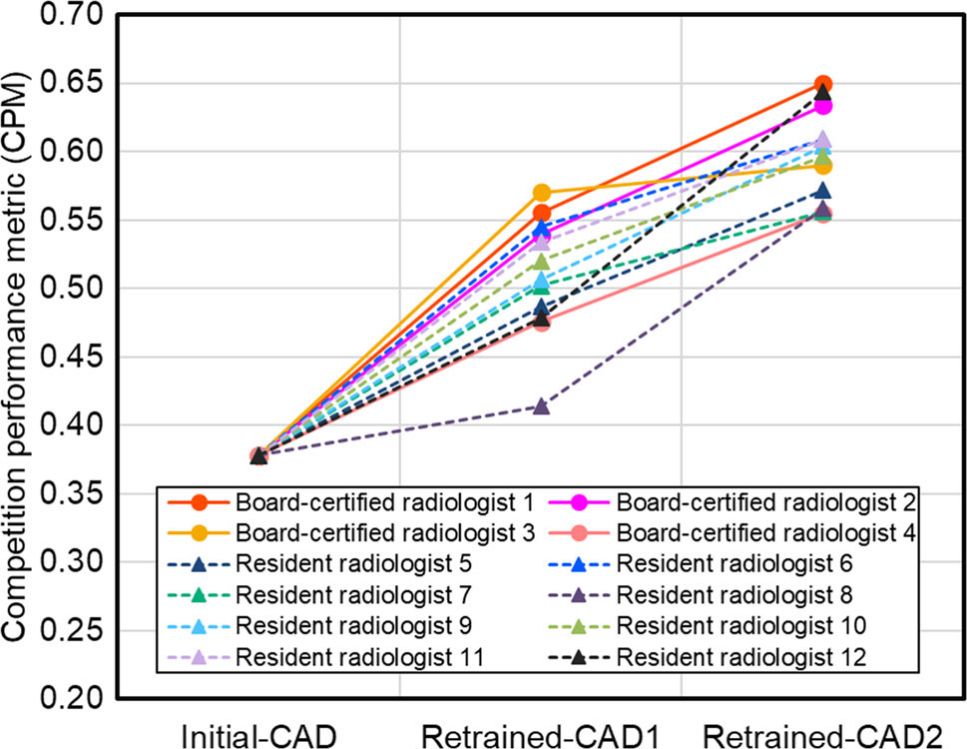
Performance change after retraining using the annotated data from each radiologist in the cerebral aneurysm detection

Figures 7 and 8 show the performance changes after retraining using the annotated data from each radiologist for lung nodule and cerebral aneurysm detection, respectively. The changes in performance after retraining varied depending on the annotating radiologist. In numerous cases, the CPM increased with the amount of training data. In lung nodule detection, the performance of Retrained-CAD1 or Retrained-CAD2 was sometimes degraded compared to that of the Initial-CAD.

**Fig. 9:**
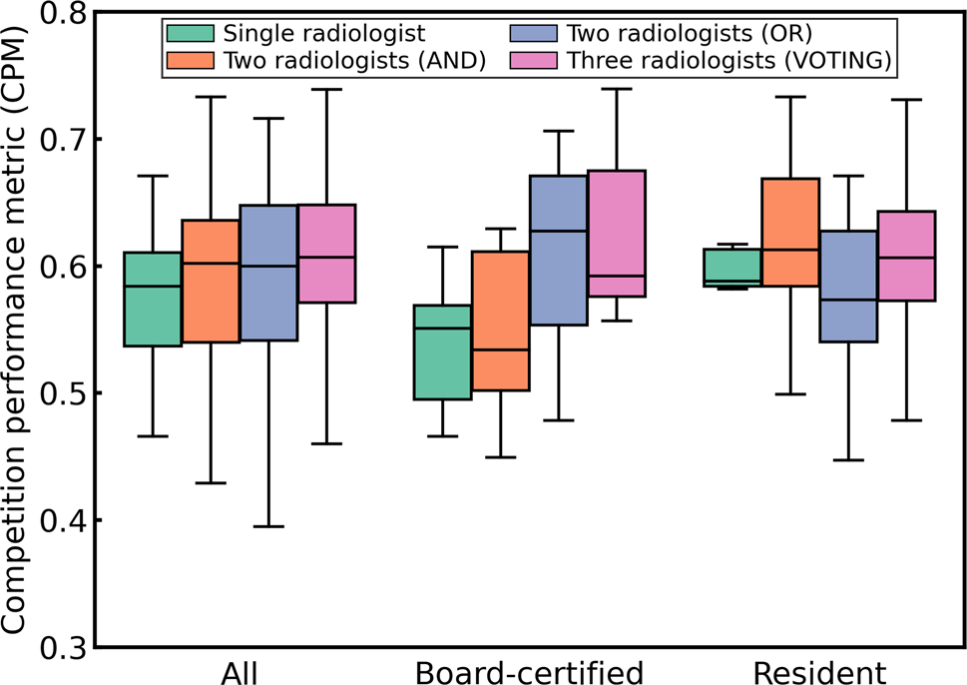
Performance of integrating annotations by multiple radiologists for lung nodule detection. There was no significant difference in the Steel–Dwass rank sum test result

**Fig. 10:**
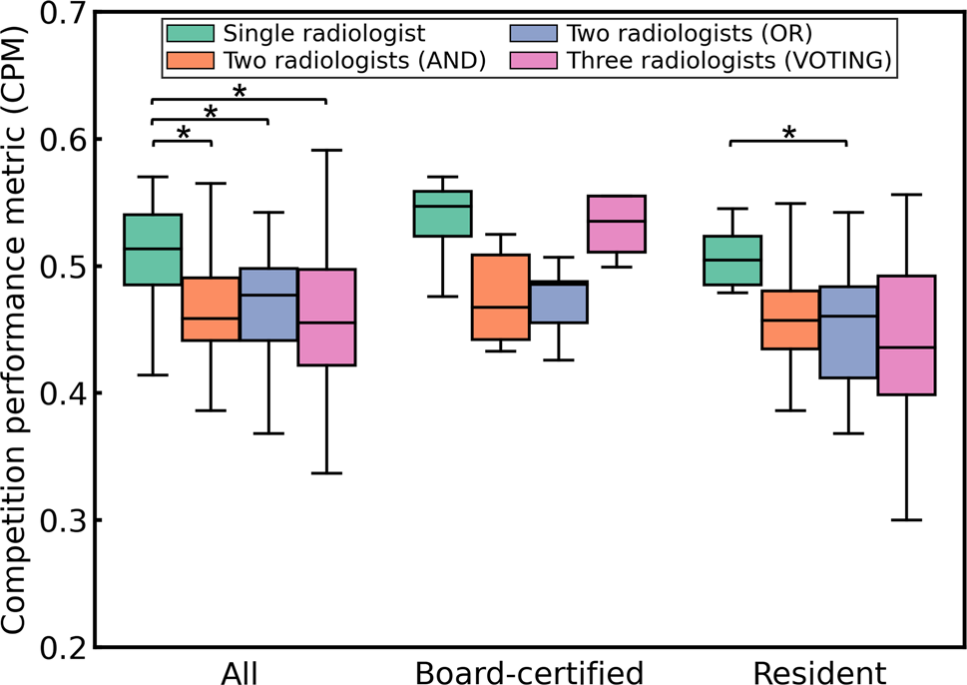
Performance of integrating annotations by multiple radiologists for cerebral aneurysm detection. *Indicates a *p* value of less than 0.05

Figure 9 shows the performance of integrating annotations by multiple radiologists for lung nodule detection. The CPMs for retraining using integrated annotations from multiple radiologists tended to be higher than those for retraining using annotations from a single radiologist regardless of the group of annotators. However, there was no significant difference in the Steel–Dwass test results. Figure 10 shows the performance of integrating the annotations by multiple radiologists for cerebral aneurysm detection. The CPMs for retraining using integrated annotations from multiple radiologists were lower than those for retraining using annotations from a single radiologist regardless of the group of annotators. Moreover, in the groups of all radiologists and resident radiologists, significant differences were observed in the Steel–Dwass test results between multiple radiologists and single radiologist.

## Discussion

We experimentally showed that the performance of the CAD software after retraining varied among the annotating radiologists. In addition, for some radiologists, who were Board-certified radiologists 1, 2, and 3 and Resident radiologist 7 in lung nodule detection (Fig. 7), the performance of the CAD software after retraining degraded compared with that of Initial-CAD. This can be attributed to personal tendencies in image diagnosis by annotating radiologists. However, as this was observed by both board-certified radiologists and resident radiologists, it cannot be directly related to the radiologists’ years of experience in image interpretation.

The factor contributing to the performance difference of Retraining-CAD1 among annotators (Figs. 7 and 8) is related to the well-known problem of label noise [28, 29] including uncertainty and inconsistency, because the annotation environment for the Retraining1 subset, including the referenced CAD software, is identical. Frénay and Verleysen [28] summarized the taxonomy of label noise in detail. Training machine learning models with label noise are problematic because they can easily overfit corrupted labels, resulting in a lack of generalizability when evaluated on a separate test set. Several research groups have reported methods for handling noisy labels in medical image analyses [30–33]. Xue et al. [30] introduced a global and local representation-guided co-training strategy without refining or relabeling noisy labeled data. Khanal et al. [32] examined the effectiveness of using a self-supervised pretraining approach to improve robustness against noisy labels in a medical image classification task. Penso et al. [33] proposed a calibration procedure for a classification model based on the fact that the confusion matrix of noisy labels can be expressed as the matrix product of the confusion matrix of clean labels and label noise. The performance of retrained CAD software can be improved by applying these methods.

Retraining using annotations integrated from multiple radiologists resulted in different trends in performance depending on the target CAD software (Figs. 9 and 10). Notably, for cerebral aneurysm detection, the performance after retraining using integrated annotations was inferior to that obtained using annotations from a single radiologist. This is not only due to variability in annotations among different radiologists but also depends on the detection sensitivity of the radiologists to the target lesions. According to a preliminary study by our group, the detection sensitivities of the radiologists were 77.4% for pulmonary nodules (5 mm or more in diameter) [34] and 64% for cerebral aneurysms [35]. Although there is no significant difference in performance among the ways of integrating annotations across any target CAD software or group of annotators, in the groups of board-certified radiologists, VOTING showed a tendency to be superior among the three ways for integrating annotations (Figs. 8 and 9). Among the three ways of integrating annotations, AND and OR are simple integrations of the two annotators. By contrast, VOTING is similar to the annotation procedure for the initial training and test subsets, in which a third radiologist resolves any discrepancies after the first two have made their annotations. Abdalla et al. stated that although majority voting is the most commonly used annotation method, it may not be the most suitable [36]. For instance, in the case of screening software, it may be worthwhile considering using a labeling method that maximizes sensitivity, such as OR. If agreement among annotators is desired, adjudicative labeling methods [37] can improve agreement. In addition, Abdalla et al. noted that when the "hard" labeling method by majority voting generates false certainty or noise, it should be expressed using "soft" labels instead [36]. Consequently, integrating annotations from multiple radiologists does not necessarily enhance the quality of the annotations and remains an open problem. We plan to apply soft labels for retraining using annotations integrated from multiple radiologists.

In the development of CAD software using machine learning, it is desirable to improve the performance by repeating the retraining at appropriate intervals. However, as shown by the results of this study, there is a possibility that performance may degrade after retraining. The management of CAD software after retraining remains an open problem, that is, how to monitor performance changes after retraining, and when and how to intervene when performance decline is suspected. Establishing well-defined quality assurance procedures is necessary to monitor the performance of CAD software through retraining. Furthermore, when continuous learning [38] is applied, humans cannot monitor continuously. Therefore, semi-automated or fully automated tools are essential for monitoring the quality and consistency of CAD software after retraining.

Our study had several limitations. First, the lesions were annotated as spherical ROIs to reduce the burden on the annotating radiologists. The results may differ if different types of annotations are used, such as contour drawings and pixel-by-pixel paintings. Second, the annotations from multiple radiologists were automatically integrated. The results may differ from the integration by the consensus of radiologists. Third, annotations for the initial training and test subsets of each CAD software were conducted by three board-certified radiologists. In this case, the uncertainty of disagreement [31], a common type of label noise in medical images, has become a problem. This uncertainty is evident from the results shown in Figs. 7 and 8. To reduce this uncertainty, Drukker et al. stated that testing model performance should not only focus on testing against a variety of independent datasets but also, if possible, against an independent pool of annotators [39]. Tachibana et al. proposed that the decision of each annotator is estimated using the machine learning model and a "virtual conference" to achieve consensus from those results [17]. This method could be used as a potential solution to this problem.

## Conclusion

We investigated the changes in performance in the retraining of the CAD software by adding cases stratified by the years of experience of the radiologists who performed the annotation. From our results, we found no direct correlation between the performance and years of experience of the radiologists, although the performance of the CAD software after retraining varied among the annotating radiologists. In addition, retraining using annotations integrated from multiple radiologists may lead to decreased performance compared to that from a single radiologist.
